# Sea Cucumber and Blueberry Extracts Suppress Inflammation and Reduce Acute Lung Injury through the Regulation of NF-κB/MAPK/JNK Signaling Pathway in Lipopolysaccharide-Treated C57BL/6 Mice

**DOI:** 10.3390/molecules29071511

**Published:** 2024-03-28

**Authors:** Oladapo F. Fagbohun, Wasitha P. D. W. Thilakarathna, Juan Zhou, Christian Lehmann, Guangling Jiao, H. P. Vasantha Rupasinghe

**Affiliations:** 1 Department Plant, Food, and Environmental Sciences, Faculty of Agriculture, Dalhousie University, Truro, NS B2N 5E3, Canada; oladapo.fagbohun@wilmington.edu (O.F.F.); wasitha@dal.ca (W.P.D.W.T.); 2 Department of Biology, Center for Agriculture and Sciences, Wilmington College, 1870 Quaker Way, Wilmington, OH 45177, USA; 3 Departments of Anaesthesia, Pain Management and Perioperative Medicine, Physiology and Biophysics, Faculty of Medicine, Dalhousie University, Halifax, NS B3H 4R2, Canada; juan.zhou@dal.ca (J.Z.); chlehmann@gmail.com (C.L.); 4 Department of Process Engineering and Applied Science, Faculty of Engineering, Dalhousie University, Halifax, NS B3H 4R2, Canada; guangling.jiao@gmail.com; 5 Department of Pathology, Faculty of Medicine, Dalhousie University, Halifax, NS B3H 4R2, Canada

**Keywords:** *Cucumaria frondosa*, *Vaccinium angustifolium*, ALI, cytokine, inflammation, NF-κB, MAPK

## Abstract

Acute lung injury (ALI) represents a life-threatening condition with high morbidity and mortality despite modern mechanical ventilators and multiple pharmacological strategies. Therefore, there is a need to develop efficacious interventions with minimal side effects. The anti-inflammatory activities of sea cucumber (*Cucumaria frondosa*) and wild blueberry (*Vaccinium angustifolium*) extracts have been reported recently. However, their anti-inflammatory activities and the mechanism of action against ALI are not fully elucidated. Thus, the present study aims to understand the mechanism of the anti-inflammatory activity of sea cucumber and wild blueberry extracts in the context of ALI. Experimental ALI was induced via intranasal lipopolysaccharide (LPS) instillation in C57BL/6 mice and the anti-inflammatory properties were determined by cytokine analysis, histological examination, western blot, and qRT-PCR. The results showed that oral supplementation of sea cucumber extracts repressed nuclear factor kappa B (NF-κB) and mitogen-activated protein kinase (MAPK) signaling pathways, thereby downregulating the expression of interleukin (IL)-1β, IL-6, and tumor necrosis factor (TNF) in the lung tissue and in the plasma. Wild blueberry extracts also suppressed the expression of IL-4. Furthermore, the combination of sea cucumber and wild blueberry extracts restrained MAPK signaling pathways by prominent attenuation of phosphorylation of NF-κB, c-Jun N-terminal kinase (JNK), and extracellular signal-regulated kinase (ERK) while the levels of pro-inflammatory cytokines were significantly suppressed. Moreover, there was a significant and synergistic reduction in varying degrees of ALI lesions such as distorted parenchyma, increased alveoli thickness, lymphocyte and neutrophil infiltrations, fibrin deposition, pulmonary emphysema, pneumonia, intra-alveolar hemorrhage, and edema. The anti-inflammatory effect of the combination of sea cucumber and wild blueberry extracts is associated with suppressing MAPK and NF-κB signaling pathways, thereby significantly reducing cytokine storm in LPS-induced experimental ALI.

## 1. Introduction

Acute lung injury (ALI) is a significant source of mortality and morbidity characterized by acute respiratory failure with acute onset of bilateral pulmonary infiltration alongside hypoxemia without evidence of hydrostatic pulmonary edema [1,2,3]. The pathogenesis of ALI can be explained by injury to both the alveolar epithelium and vascular endothelium [4], resulting in acute respiratory distress syndrome (ARDS) [5]. ARDS is common with over 200,000 incidences per year worldwide and there is an unmet medical need for interventions and treatment [6]. Recently, ARDS and ALI were associated with the COVID-19 pandemic [7]. Acute pulmonary inflammation causes disruption of the lung epithelial and endothelial barriers [8]. Moreover, biomarkers found on the epithelium and endothelium that are involved in the coagulation and inflammatory cascades predict morbidity and mortality in ALI [9,10]. Even in patients who survive ALI, there is evidence that their long-term quality of life is adversely affected. The cellular characteristics of ALI include excessive transepithelial neutrophil migration into the alveolar and interstitial spaces, loss of alveolar–capillary membrane integrity, the release of pro-inflammatory and cytosolic mediators such as interleukin (IL-6), IL-1β, and tumor necrosis factor (TNF), and upregulation of nuclear factor kappa B (NF-κB) and mitogen-activated protein kinases (MAPK) signaling pathways [11,12]. Several studies have shown that, following infection or injury to the lung tissues, plasma levels of pro-inflammatory cytokines can increase significantly (cytokine storm), leading to dysfunction and subsequent failure of multiple organs [13,14,15]. Therefore, there is a need for urgent intervention for the treatment of ALI and the prevention of systemic inflammation seen in ALI patients.

Previous treatment strategies are based on both ventilatory and non-ventilatory approaches [16,17]. To date, the most significant advances have been associated with improved ventilator management [1,18]. Several large clinical trials studying pharmacologic strategies have not been effective in reducing mortality [19,20]. Optimal fluid management for ALI patients has been controversial due to the incidence of non-pulmonary organ failure with restrictive fluid management, specifically, renal failure [21,22]. Recent advances in the understanding of the pathophysiology of ALI have led to several experimental investigations of potential pharmacological treatments [23]. A promising approach is the use of 3-hydroxy-3-methylglutaryl-CoA synthase and 3-hydroxy-3-methylglutaryl-CoA reductase (HMG-CoAR) inhibitors (statins), which possess significant antioxidant, immunomodulatory, and anti-inflammatory effects. However, it is not clear how this treatment strategy translates to the human ALI/ARDS population despite the decreased severity of sepsis and mortality from several observational studies [24]. Other novel treatment approaches involve the use of bone-marrow-derived mesenchymal stem cells (MSC) which possess the ability to differentiate into many types of cells (vascular endothelium and alveolar epithelium) with a resultant reduction in the severity of ALI and delivery of potential therapies via aerosol to the distal air spaces of the lungs. Nevertheless, translating these experimental studies to phase I and II clinical trials has not been implemented [25,26,27].

Natural products and food bioactives with low toxicity and minimal side effects have been postulated as novel alternative ALI treatments [15,23]. Two such natural products are sea cucumber (*Cucumaria frondosa*) [28,29] and wild blueberry (*Vaccinium angustifolium*) [30]. *C. frondosa* contains many nutrients and bioactive compounds, namely amino acids, cerebrosides, collagen, polysaccharides, polyphenols, and saponins, which demonstrate its unique pharmacological and biological activities. Bioactives of *C. frondosa* can act as anticancer, antimicrobial, antioxidant, anti-angiogenic, antidiabetic, and anti-inflammatory agents [28,29]. The mechanism of action of saponins (primarily frondoside A) found in *C. frondosa* is postulated to include the inhibition of the production of pro-inflammatory cytokines such as tumor necrosis factor-alpha (TNF-α), interleukin (IL)-1β, and enzymes cyclooxygenases (COX-1 and COX-2) by PI3K/AKT/ERK1/2/p38 MAPK, RAC/CDC42 PAK1, and LXR-β signaling pathways [29,31,32,33,34]. Therefore, natural molecules of *C. frondosa* that can reduce cytokine storms as well as the levels and/or concentrations of cyclooxygenases are potential drug candidates for ALI. On the other hand, *V. angustifolium* extracts have been found to contain polyphenols, especially anthocyanin, with antioxidant and anti-inflammatory properties [30]. However, there is limited knowledge of these natural products as potential treatments against ALI. Therefore, this study was designed and conducted to investigate the anti-inflammatory properties of *C. frondosa* extract and *V. angustifolium* extract in an experimental ALI by testing the plasma and tissue concentrations of inflammatory cytokines and the complement and analyzing the signaling pathways implicated in acute inflammation in the development of ALI. In addition, the synergistic effects of the combination of *C. frondosa* extract and *V. angustifolium* extract were also investigated in the treatment of ALI.

## 2. Results

### 2.1. Body Weight of Experimental Mice

At the start of the experiment, the mean body weights of the mice in the experimental groups were similar, having an average of 25.15 g/mouse. After the treatment, the body weights of group 3 (Table 1) were decreased when compared with the initial body weights. Furthermore, groups 4 and 5 had 1.84 and 3.85% increases in their body weights, respectively. It should be noted that the mice were fed with the same diet; however, the diet of group 3 was supplemented with the extract of sea cucumber whereas group 4 was supplemented with the extract of wild blueberry. Moreover, group 5 mice had their diet supplemented with a mixture of sea cucumber and wild blueberry extracts. The weight gain in group 4 mice paralleled that of the control group, while the reduction in body weight of group 3 mice showed that sea cucumber extracts do not lead to an increase in body weight. By contrast, the weight gain in group 5 experimental mice could be a result of the addition of wild blueberry extracts.

### 2.2. Effect of Sea Cucumber and Wild Blueberry Extracts on IL-6, IL-1β, Vascular Endothelial Growth Factor (VEGF), and Epidermal Growth Factor (EGF) Levels

**Figure 1:**
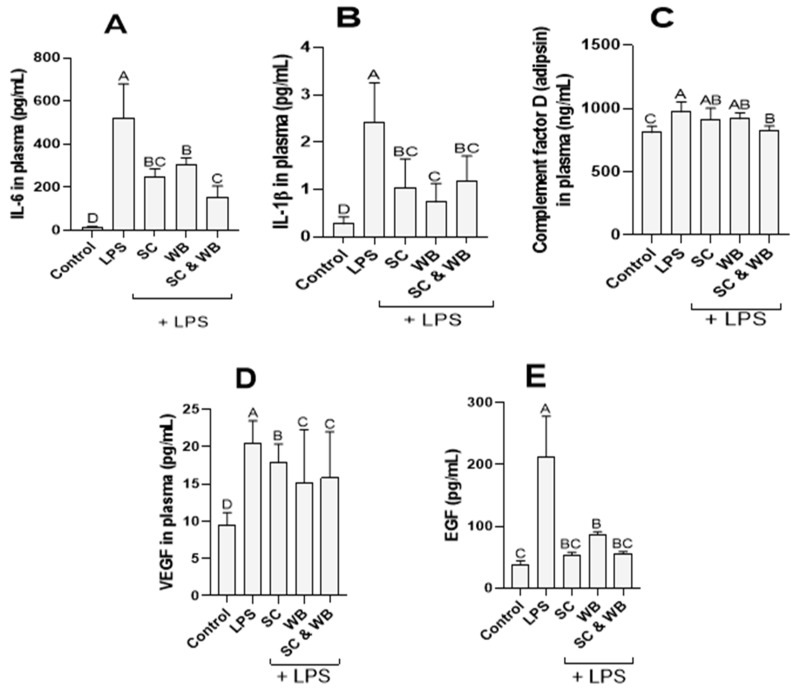
Plasma levels of interleukin (IL)-6 (**A**), IL-1β (**B**), complement factor D (**C**), vascular endothelial growth factor (VEGF) (**D**), and endothelial growth factor (EGF, (**E**)) in the mice, treated with intranasal administration of saline (Control), or LPS (LPS), or LPS with oral supplementations of the body parts (BP) of sea cucumber (SC), wild blueberry (WB), or combination of sea cucumber and wild blueberry (SC (BP) and WB). Proteins were quantified using Luminex assay. Means that do not share a similar letter (A–D) are significantly different. Significance was taken at *p* ≤ 0.05 when compared with the control; *n* = 10.

The effects of sea cucumber and wild blueberry extracts on the levels of cytokines were examined in vivo. Plasma samples from experimental mice revealed that LPS significantly stimulated the production of IL-1β and IL-6 at the 6 h timepoint, leading to cytokine storm (Figure 1). It was also observed that complement factor D and VEGF levels were significantly elevated following ALI induction. The protein levels were significantly reduced in animals supplemented with the combined extracts of sea cucumber and wild blueberry (Figure 1).

### 2.3. Sea Cucumber and Wild Blueberry Attenuate Histopathological Signs of Experimental ALI

**Figure 2:**
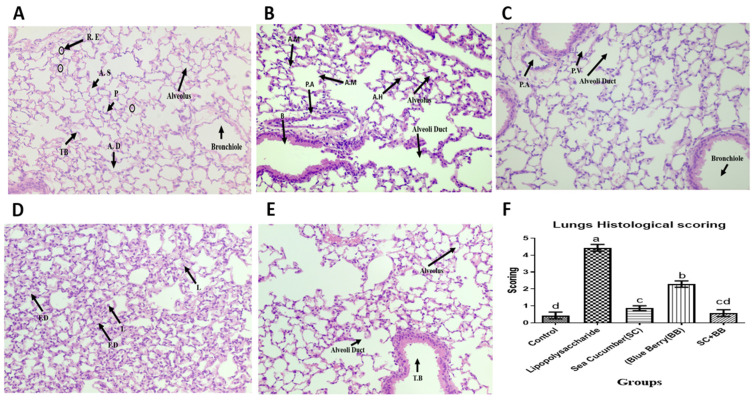
Representative light micrographs of H and E-stained lung sections of nasal saline or LPS-treated mice with or without oral supplementations. (**A**) Control group; (**B**) LPS; (**C**) LPS + SC; (**D**) LPS + WB; (**E**) LPS + SC + WB; (**F**) bar graph of mean comparison of scores. Abbreviations: **LPS**, lipopolysaccharides; **SC**, extract of body parts of sea cucumber; **WB**, extract of wild blueberry; **A.D**, Alveoli Duct; **A.S**, Alveolar space; **T.B**, Terminal Bronchi; **P.A**, Pulmonary Endothelium; **L**, Lymphocyte infiltration; **F.D**, Fibrin Deposition; **A.M**, Alveoli Macrophages; **A.H**, Alveoli Hemorrhage; **B**, Bronchiole. In Figure 2F, means that do not share a similar letter (a–d) are significantly different. Significance was taken at *p* ≤ 0.05 when compared with the control; *n* = 10.

We examined the histopathological changes in lung tissues of ALI animals following diet supplementation with *C. frondose* extracts and *V. angustifolium* extracts. The lung tissues were collected and evaluated for inflammatory activities six hours after ALI induction. The control mice (A) showed intact morphological features of respiratory epithelium comprising of the terminal bronchiole, leading into the alveoli duct (Figure 2). The terminal bronchiole (TB) of mouse lungs leads into the alveolar duct (AD) from where it branches into the alveoli sacs and terminates in the alveoli. The terminal bronchiole consists of simple cuboidal epithelium and a layer of smooth muscle fiber. This cuboidal ciliated epithelium of the terminal bronchiole loses its cilia distally as it becomes low cuboidal running into less luminal diameter alveoli ducts from where it terminates into the alveoli predominantly lined by flattened type I cells and cuboidal type II cells. A few resident macrophages (dust cells) are also notable within the respiratory epithelium. As seen in Figure 2B, LPS-treated mice show varying degrees of lesions such as distorted parenchyma, increased alveoli thickness, lymphocyte and neutrophil infiltration, fibrin deposition, pulmonary emphysema, pneumonia, intra-alveolar hemorrhage, and edema. Groups C, D, and E are the intervention groups, consisting of sea cucumber, blueberry, and a combined administration of the two, respectively. Histological observations in the pretreated groups revealed a reduction to complete absence of the observed lesions when compared with the LPS-treated mice.

### 2.4. Supplementation of Sea Cucumber and Wild Blueberry Extracts Attenuated LPS-Induced Inflammation via NF-κB and MAPK Signaling Pathways

**Figure 3:**
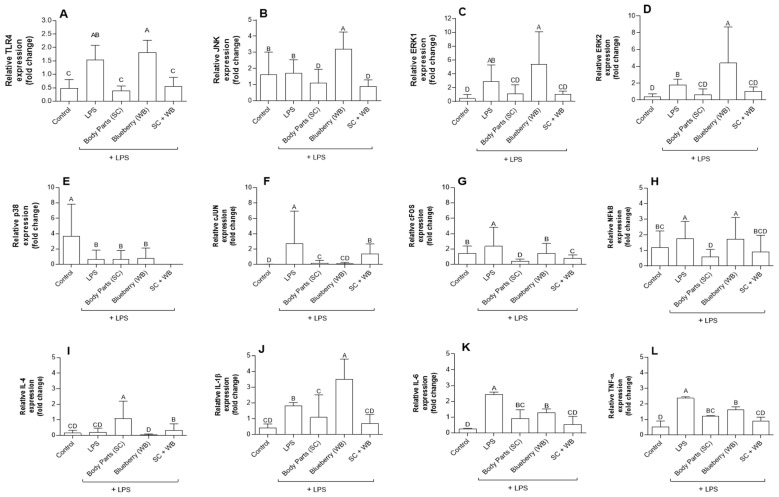
Fold changes of the expression of selected genes (mRNA) of liver samples from nasal saline or LPS-treated mice with or without oral supplementations. The diets of mice were supplemented with the extracts of sea cucumber or wild blueberry, separately or in combination. The genes investigated are toll-like receptor 4 (TLR-4, (**A**)), c-JUN N-terminal kinases (JNK, (**B**)), serine/threonine protein kinase (ERK)1/2 (**C**,**D**), mitogen-activated protein kinase p38 (p38, (**E**)), c-Jun proto-oncogene (cJUN, (**F**)), proto-oncogene c-Fos (c-FOS, (**G**)), nuclear factor kappa B (NF-κB, (**H**)), interleukin (IL)-4 (**I**), IL-1β (**J**), IL-6 (**K**), and tumor necrosis factor (TNF) (**L**). Glyceraldehyde-3-phosphate dehydrogenase (GAPDH) and β-actin were used as the internal control. Data were presented as mean ± SD. Means that do not share a similar letter (A–D) are significantly different. Significance was taken at *p* ≤ 0.05 when compared with the control; *n* = 10.

To investigate the mechanism of the anti-inflammatory effect of the extracts, we examined the mRNA expression on the lung tissue in the inflammatory signaling pathways. Sea cucumber extracts inhibited transcript levels of NF-κB and MAPK, leading to downregulation of IL-1β, IL-6, and TNF expressions, suggesting that sea cucumber extracts act as an anti-inflammatory agent since these cytokines are known as pro-inflammatory mediators (Figure 3). Upon LPS stimulation, cytokines-mediated inflammatory cell death is observed [35,36]. In our study, LPS-induced ALI is evidenced by overexpression of NF-κB and MAPK kinase pathways. However, wild blueberry extracts inhibited IL-4 expression (Figure 3), suggesting that wild blueberry extracts can act as anti-inflammatory agents. Therefore, the combination of sea cucumber and wild blueberry extracts can synergistically act as anti-inflammatory agents.

### 2.5. Sea Cucumber and Wild Blueberry Extracts Reduced NF-κB and MAPK Expression While the Combined Extracts Increased JNK Expression

**Figure 4:**
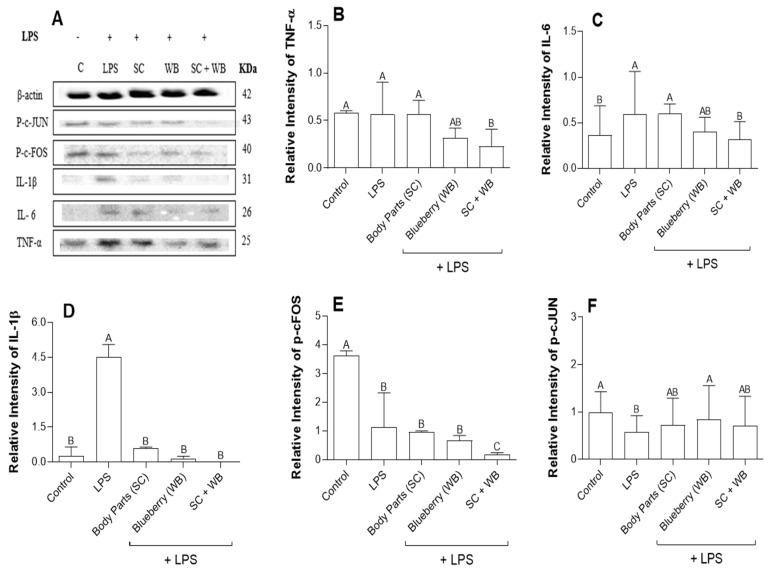
Protein levels of the tumor necrosis factor (TNF) (**B**), interleukin (IL)-6 (**C**), IL-1β (**D**), p-cFOS (**E**) and p-cJUN (**F**). Protein levels were quantified using Western blotting (**A**). The diets of C57BL/6 mice were supplemented with the extracts of *C. frondosa* extracts and *V. angustifolium* separately or in combination. Data were presented as mean ± SD and normalized by β-actin from three independent studies. Means that do not share a similar letter (A–C) are significantly different. Significance was taken at *p* ≤ 0.05 when compared with the control; *n* = 10.

**Figure 5:**
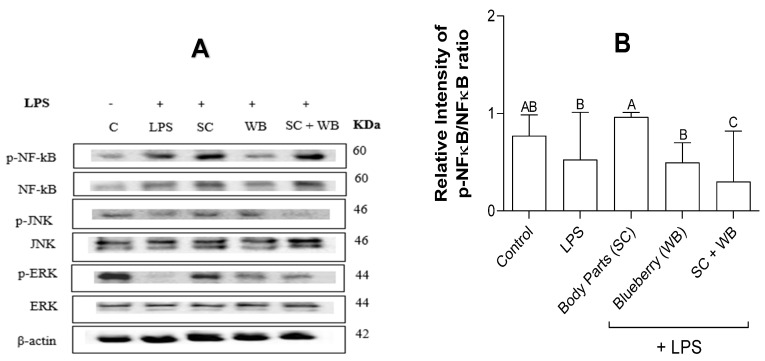

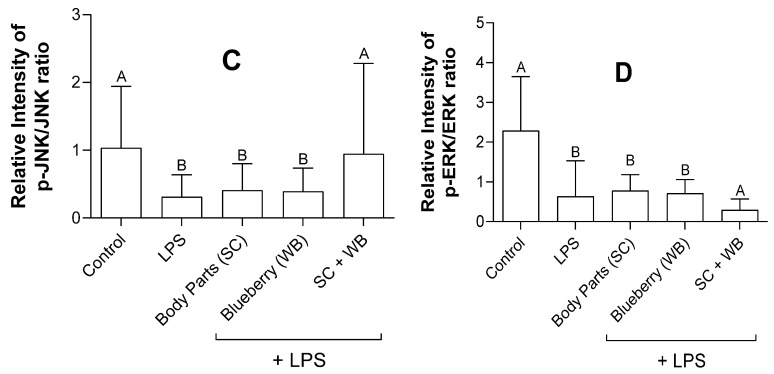
Protein levels of phosphorylated and non-phosphorylated nuclear factor kappa B (NF-κB, (**B**)), JNK (**C**), and ERK (**D**). Protein levels were quantified using Western blotting. β-actin was used as an internal control (**A**). The diets of C57BL/6 mice were supplemented with the extracts of *C. frondosa* extracts and *V. angustifolium*, separately or in combination. Data were presented as mean ± SD and normalized by β-actin. The ratio of phosphorylated and non-phosphorylated proteins was calculated from three independent studies. Means that do not share a similar letter (A–C) are significantly different. Significance was taken at *p* ≤ 0.05 when compared with the control; *n* = 10.

**Figure 6:**
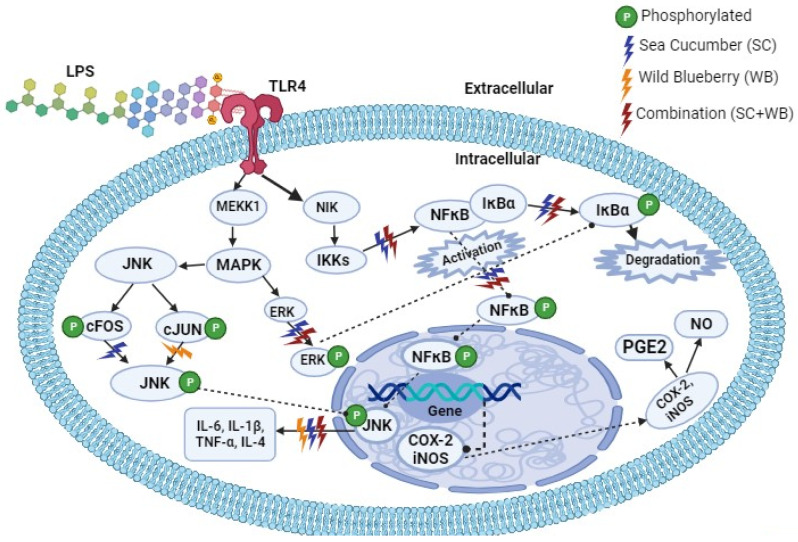
The protective effects of sea cucumber and wild blueberry extracts on acute respiratory distress syndrome (ARDS) model of acute lung injury (ALI) and the underlying mechanisms. Schematic view of the TLR4 and signaling pathway for *C. frondosa* extracts and *V. angustifolium* extracts as well as their combinations in the metabolism of LPS-induced infection. The extracts of *C. frondosa* extracts and *V. angustifolium* act by repressing NFκB and MAPK signaling pathways. The dotted lines represent postulated cell signaling pathways. TLR4, toll-like receptor 4; MAPK, mitogen-activated protein kinase; LPS, lipopolysaccharide; JNK, c-JUN N-terminal kinases; ERK, serine/threonine protein kinase; COX-2, cyclooxygenase-2; NF-κB, nuclear factor kappa B; iNOS, nitric oxide synthase; NO, nitric oxide; PGE2, prostaglandin E2, IL-1β, IL-4, IL-6, Interleukins; TNF-α, tissue necrosis factor-alpha.

Consistent with the qRT-PCR results (Figure 3), *C. frondosa* extracts and *V. angustifolium* extracts reduced the expression of NF-κB and mitogen-activated protein kinases (MAPK) expression studied by Western blotting analysis (Figure 4). Previous studies have demonstrated that NF-κB and MAPK play a vital role in the pro-inflammatory polarization of macrophages [37,38]. Therefore, we examined the effects of the extracts on the expression of these proteins. The extracts of *C. frondose* extracts and *V. angustifolium* significantly reduced LPS-induced phosphorylation of NF-κB, JNK, and ERK proteins, suggesting the inhibitory roles of these extracts in either pro- and anti-inflammatory polarization (Figure 5). Interestingly, the combination of *C. frondosa* extracts and *V. angustifolium* extracts significantly increased the phosphorylation of JNK, thereby resulting in increased phosphorylation of cFOS and cJUN, which have been widely postulated as the reason for increased transcriptional factors leading to the inhibition of IL-1β, IL-6, and TNF-α. These findings suggest the combination of extracts play a role as anti-inflammatory agents (Figure 6).

## 3. Discussion

Life-threatening systemic inflammation (cytokine storm) can be triggered by various pathological conditions [39,40]. We used LPS to induce ALI in C57BL/6 mice. The pathogenesis of ALI begins with inflammatory damage to the alveolar–capillary membrane [4]. Like any other form of acute inflammation, the permeability of the surrounding vasculature increases. The LPS challenge targeted the lung in our study, and as the lung permeability rises, protein-rich pulmonary edema fluid is drawn into the lungs, ultimately leading to respiratory insufficiency [41]. This is similar to what was reported during the SARS-CoV and MERS-CoV infections. ALI is considered to be the hallmark of the clinical consequence of SARS-CoV-2 by the immune system [42]. From the results of this study, it was revealed that there were elevated levels of inflammatory biomarkers that led to cytokine storm in LPS-challenged mice without oral treatment of either sea cucumber extracts, wild blueberry extracts, or their combination.

IL-6 is promptly and transiently produced in response to infections. It has a pathological effect on chronic inflammation and autoimmunity [43]. In our study, LPS-challenged mice had higher levels of IL-6. Although there were elevated levels of IL-6 in groups C, D, and E (Figure 1), oral supplementation of the extracts of body parts (BP) of sea cucumber (SC), wild blueberry (WB), or their combination showed a significant reduction in the production of IL-6. Moreover, a significant reduction in ALI symptoms (Figure 2) and expression of proinflammatory biomarker genes (Figure 3) is observed in group E, which is the combination of polyphenols (anthocyanin in wild blueberry) and saponins in the sea cucumber. TNF is a potent paracrine and endocrine mediator of inflammation [44]. It regulates the growth and differentiation of a wide variety of cell types. Elevated levels of TNF have been linked to many diseases, including COVID-19 [45,46]. From the results of our study, there appears to be a surge in the levels of TNF in LPS-challenged mice. Furthermore, the levels of TNF were reduced after the dietary supplementation of sea cucumber and wild blueberry extracts (Figure 3). Interestingly, the effects of oral supplementations were observed in group 3 alone and also in combination. It is known that TNF induces inflammation by triggering the production of several immune system molecules, including IL-1 and IL-6 [47]. Furthermore, TNF and IL-1β activate several transcription factors to produce IL-6 [48]. This means that TNF and IL-6 are positively correlated with each other. Hence, both IL-6 and TNF are pro-inflammatory cytokines produced in response to infections such as LPS challenge. The reduction in their production is a goal for the treatment of several autoimmune diseases. Moreover, natural IL-6 and TNF blockers are postulated to be novel drug leads in the treatment of inflammation. This is observed from the results of this study.

The complement system plays a vital role in inflammation by regulating various steps of an inflammation response [49]. The system is made up of a large number of distinct plasma proteins that react with one another to opsonize pathogens and induce a series of inflammatory responses that help to fight infections [50]. Several complement proteins are proteases that are themselves activated by proteolytic cleavage. Also, the elevated levels of some other complement proteins are signals for the responses of some important cytokines [51]. The functions of the complement system in innate host defense are accomplished through three broad effector pathways: lysis, inflammation, and opsonization/phagocytosis [52]. The complement proteins studied here are EGF, VEGF, and complement factor D (Adipsin) (Figure 1). It is known that IL-6 induces excess production of VEGF and EGF, leading to enhanced angiogenesis and increased vascular permeability which are pathological features of inflammation lesions seen in synovial tissues of rheumatoid arthritis (RA) or edema of remitting seronegative symmetrical synovitis with pitting edema (RS3PE) syndrome [53].

ALI results from direct and indirect lesions to the parenchyma of the lungs [4,54], and the mortality rate has increased exponentially to as much as 40% due to the slow progress in identifying the processes responsible for the disease’s development and progression [55]. Inhalational instillation of LPS induces experimental ALI. It is a well-established model of ALI in animals with documented closely related symptoms in humans [56,57]. In our study, LPS-induced acute lung injury showed patterns of distorted parenchyma, increased alveoli thickness, intra-alveolar hemorrhage, and pulmonary edema, as well as severe acute pulmonary inflammation evidenced by lymphocyte and neutrophils infiltration, and fibrin deposition. Asti and Ruggieri [58] and Zou and Dong [59] reported similar lesions in LPS-induced ALI. However, pretreatment of mice with sea cucumber and wild blueberry protected the lung parenchyma from epithelial and endothelial cell damage and preserved the integrity of the blood–air barrier and air–alveolar interface. This could be due to the protective effect of sea cucumber on soft tissues reported earlier [33]. Moreover, there is evidence that saponins which are meant for the chemical defenses of sea cucumbers play an important role in the alleviation of inflammation. Our previous study also discovered more saponins in the internal organs than the body walls, which agreed perfectly with the previous studies of Bahrami and Franco [60], Bahrami, Zhang [61], Bahrami, Zhang [62], Bahrami, Zhang [63], and Bahrami and Franco [64].

Neutrophils have been implicated to be involved in the pathogenesis of ALI [37,65]. IL-8 and other pro-inflammatory cytokines have been identified in the migratory activities of neutrophils, which in turn induces migration through the alveolar–capillary barrier, thereby resulting in the accumulation of neutrophils in the alveolar space [66]. This explains the accumulation of neutrophils observed in the LPS-only treated group. However, administration of the extracts of sea cucumber and blueberry blocked the progression of the inflammatory response by which acute lung injury would have been achieved. This leads to the anti-inflammatory potential of the bioactives of sea cucumber and blueberry which has been widely reported in several other studies [28].

Blueberries have been reported to contain polyphenols that have anti-oxidative, anticancer, and anti-inflammatory effects [30]. Blueberry polyphenols exhibit their anti-inflammatory potential by interrupting oxidative stress via the anti-oxidative stress pathway, accompanied by promoting arachidonic acid metabolism. This leads to the accumulation of phagocytic cells at the inflammatory loci, mediated by proinflammatory factors and the release of large quantities of reactive oxygen species which could possibly result in lipid peroxidation and promote the release of the lysosome, leading to an eventual reduction in the release of various inflammatory mediators [67]. Reactive oxygen species serve as an intermediary in the transfer of energy and participate in many functional and biomedical reactions in the human body. The balance brought about by free radicals is important for maintaining stable health and homeostasis. Recently, antioxidant/oxidant assessments have been used to deduce oxidative homeostasis, and it is directly proportional to the health of the individual [68]. Sea cucumbers possess anti-inflammatory properties with effects on soft tissue repairs [33]. They contain omega-3 fatty acids such as eicosapentaenoic and docosahexaenoic acids, whose action inhibits the synthesis of prostaglandin by suppressing the expression of cyclooxygenase-2 and 5-lipoxygenase under inflammatory conditions [29,69]. The heparin analogs of sea cucumber, *Styela plicata* (pleated sea squirt), have also been reported to possess an anti-inflammatory effect on colon inflammation in rat models [34,70]. In agreement with the literature, the diet of experimental animals supplemented with sea cucumber and wild blueberry extracts showed a significant reduction in infiltration of macrophage and T-cells, which eventually suppressed the storms of tumor necrosis factors and other signaling pathways, such as the NF-κB and MAPK pathways, thereby inhibiting IL-1β, TNF, and IL-6. Moreover, the results of this project revealed a downregulation of the MAPK and NF-κB pathways by the tested extracts, as well as a reduction in the phosphorylation of these proteins. This explains why we have a completely preserved lung architecture in the groups pretreated with sea cucumber before LPS induction.

Upon LPS inhalation into the organism, LPS binds to Toll-like receptor (TLR) 4 at the surface of pulmonary cell membranes, thereby leading to increased cellular concentration of MAPK and other inflammatory signaling pathways (Figure 6). TLR4 acts as a homodimer together with the accessory protein, myeloid differentiation factor 2 (MD-2), and mediates recognition of LPS. The binding of LPS triggers the phosphorylation of NF-κB, leading to the activation of cellular responses. The activation of the MAPK signaling pathway has been implicated as a critical step in the process of pulmonary barrier dysfunction induced by various stimuli [71]. The results of this study revealed that there was a significant reduction in NF-κB and MAPK signaling pathway activation. Sea cucumber extract and its synergistic activities with wild blueberry extracts represent potential novel and natural therapeutics for the treatment of ALI.

## 4. Materials and Methods

### 4.1. Chemicals and Reagents

Lipopolysaccharide (LPS) from *Pseudomonas aeruginosa*, ethanol, pentobarbital, and other chemicals were procured from Sigma Millipore, Oakville, ON, Canada.

### 4.2. Experimental Animals

Eleven-to-thirteen-week-old adult male C57BL/6 mice were procured from Charles River Laboratories (Spencerville, OH, USA). They were maintained in specific-pathogen-free standardized conditions at a temperature of 23 ± 2 °C and humidity of 55 ± 5% with a 12 h light/dark cycle in the Carleton Animal Care Facility of the Sir Charles Tupper Medical Building (Halifax, NS, Canada), and had ad libitum access to standard rodent chow and water. All methods and experiments were conducted in accordance with the Principles of Laboratory Animal Care of the National Institute of Health and the guidelines of the Laboratory Animal Care Committee of Dalhousie University, Halifax, NS, Canada, under protocol number 20-075.

### 4.3. Extraction of Sea Cucumber Materials and Purification of Saponins

The extraction and purification procedures were adapted from the optimization extraction model developed in our laboratory using response surface methodology (RSM) and an artificial neural network (ANN). The internal organs, also called the body parts (BP), of the sea cucumber were provided by AKSO Marine Biotech Inc., Halifax, NS, Canada. The extraction of saponins from the dehydrated internal organs was optimized using response surface methodology (RSM) with ultrasonication-assisted extraction. The optimized extraction conditions are 80% ethanol, 50 °C sonicator temperature, and 60 min exposure time. The optimized extract was concentrated using a Heidolph Hei-VAP advantage rotary evaporator (Heidolph Instruments GmbH, Schwalbach, Germany) and freeze-dried using a Dura-Stop FitsSystems^TM^ stoppering tray dryer (American Laboratories, Southport, CT, USA). Finally, the optimized extract was cleaned up using HyperSep™ glass block (SPE) manifolds (Waters Corporation, Milford, MA, USA). The SPE clean-up steps for saponin compounds involve the use of 50 mg by 1 cc Sep-Pak Vac C_18_ cartridges (Waters Corporation, Milford, MA, USA) with an SPE manifold. The resulting extracts were freeze-dried and stored at 4 °C until further analysis.

### 4.4. Extraction Procedure for Wild Blueberry

Fully mature wild blueberries were collected from a commercial farm (Brag Lumber Company) located in Highland Village, NS, Canada (45°24′17.9″ N 63°40′14.0″ W) during early August of 2022. The frozen whole berries were ground, and the resulting pulp was filtered through eight layers of cheesecloth at room temperature in a semi-dark condition. The filtrate was centrifuged at 1000× *g* for 10 min and the supernatant was stored at −20 °C overnight. The frozen filtrate was freeze-dried at −40 °C for 48 h using a Dura-Stop FitsSystems^TM^ stoppering tray dryer (American Laboratories, Omaha, NE, USA), and the dehydrated extract was stored at −80 °C until use.

### 4.5. Quantification of Frondoside A Using Ultra-High-Performance Liquid Chromatography-Electrospray Ionization-Mass Spectrometry (UPLC-ESI-MS)

Frondoside A, the major saponin compound of sea cucumber extract, was quantified using UPLC-ESI-MS. The filtered samples (2 µL) were injected into the UPLC system (Waters Acquity UPLC H-class, Waters Limited, Milford, MA, USA) equipped with a phenyl-hexyl X-Bridge column (4.6 mm × 100 mm, 5 µm) and a tandem quadrupole mass detector (Waters Quattro Micro, Waters Limited, Milford, MA, USA). The mobile phase was 0.1% formic acid in water (solvent A) and 0.1% formic acid in methanol (solvent B). The flow rate of 0.7 mL/min was set in a linear gradient mobile phase of solvent B applied at time t (min) as follows (t, B%): (0, 10%), (7, 30%), (16, 50%), (17, 50%), (18, 80%), (25, 80%), (27, 10%), and (35, 10%). The MS conditions were ESI in negative ion mode (ESI-) with a capillary voltage of 3000 V, nebulizer gas (N2) temperature of 375 °C, source temperature of 150 °C, and cone temperature of 60 °C. Frondoside A was detected (retention time of 21.9 min) using the single ion mode of *m/z* = 1312.

### 4.6. Quantification of Total Anthocyanins by the pH Differential Method

The total anthocyanins of the wild blueberry extract were estimated using the pH-differential (AOAC method 2005.02) method [72]. Ten-fold diluted extract in both pH 1 and pH 4.5 buffers was prepared in duplicate, and the absorbance was measured at 520 nm and 700 nm using a microplate reader (Tecan Infinite^®^ M200 PRO, Morrisville, NC, USA). The absorbance values (A) of samples were calculated as follows:

A = (A520 − A700) at pH 1.0 − (A520 − A700) at pH 4.5

Then, total anthocyanins were calculated as follows:

Total anthocyanins = (A × MW × DF × 1000)/(ε × L)

The molar extinction coefficient (ε); 26,900, molecular weight (MW) of petunidin-3-*O*-glucoside (P3G); 479.4 g/mol, dilution factor (DF); and path length (L) [72].

### 4.7. Diet Preparation by Palletization of Sea Cucumber and Blueberry Extracts

The mice doses of dietary supplementation of sea cucumber and wild blueberry extracts were estimated from previously reported mice studies [34,73] in which the therapeutic effective dose of triterpene glycoside (frondoside A) was 1 mg/kg body weight of mouse/day [34] while the effective dose for total anthocyanins was 48 mg (P3G equivalents, P3GE)/kg body weight of mouse/day [73]. The concentration of frondoside A in sea cucumber extract was determined by UPLC-ESI-MS, and the total anthocyanins in wild blueberry extract were determined using the pH-differential spectrophotometric method and expressed as mg P3GE/g dry weight (DW) of the extract. Based on the total anthocyanins present in the wild blueberry extract (18.95 mg P3GE/g DW) and frondoside A in sea cucumber extract (0.36 mg/g DW) of our study, the amount of sea cucumber and wild blueberry extracts required for each diet were calculated and incorporated into the mouse diet (Table 2). The sea cucumber and blueberry extracts were mixed individually or as a combination with the chow to make pellets. The mice consumed an average of 5 g of chow per day; therefore, the dried powders of the *C. frondosa* extracts and *V. angustifolium* extracts were incorporated into the chow powder, and then pellets were prepared to obtain the required experimental diets according to a previously established method [74].

### 4.8. Experimental Model of Acute Lung Injury (ALI)

C57BL/6 mice were challenged with 5 mg/kg lipopolysaccharide (LPS) via intranasal instillation to induce experimental ALI [40]. Briefly, the mice were anesthetized using 5% isoflurane in the presence of oxygen. The anesthetized mice were positioned on an induction clamp and drops of LPS were released into their right nostrils at an interval of 1 drop per 10 s count while the tongue was carefully held with a forceps to facilitate the LPS entry into the lower respiratory tract. These animals were maintained under 3% isoflurane for the entire duration of the experiment.

A total of 50 mice were randomly divided into 5 groups (10 mice in each group). The control group (the group with intranasal administration of normal saline only), the LPS group (the group with intranasal administration of LPS only), the SC group (the group with an oral supplement of *C. frondose* materials and intranasal LPS only), the WB group (the group with an oral supplement of *V. angustifolium* extracts and intranasal LPS only), and the SC + WB group (the group with intranasal LPS and oral supplements of SC and WB). The mice were given oral supplementation of *C. frondosa* extracts and *V. angustifolium* extracts daily for 7 consecutive days before intranasal LPS instillation on the 8th day. The duration for the extract administration was based on the modification of previous studies [75,76]. Six hours after LPS instillation [40], behavioral assessment was conducted, including the mobility score and body weight. The combination of *C. frondosa* extract and *V. angustifolium* extract was based on the Chou-Talalay isobologram combination method [77] where synergism was achieved at SC + WB; 25: 100 μg/mL ratio. After the 6 h time point, the mice were euthanized with pentobarbital. Blood, liver, and lung tissues were collected from the mice. The blood samples were collected using a heparin-coated 1 mL syringe, and the plasma was obtained after 20 min centrifugation at 2000× *g* and stored at −20 °C for further analysis. On the other hand, the lung tissues were prepared for histology by inflaming the lungs through the insertion of the needle containing formaldehyde into the trachea while some lung samples were kept at −80 °C for biochemical analyses.

### 4.9. Luminex^®^ Multiplex and Enzyme-Linked Immunosorbent (ELISA) Assays

Luminex kits were purchased from Bio-Techne (R&D Systems Inc., Minneapolis, MN, USA) for measuring the levels of IL-6, complement factor D (Adipsin), VEGF, and EGF in the plasma. The level of IL-1β was determined by the high-sensitivity ELISA kit purchased from Bio-Techne (R&D Systems Inc., Minneapolis, MN, USA).

### 4.10. Lung Histological and Histopathological Examination

The harvested lung tissues were fixed in 4% paraformaldehyde, embedded in paraffin, and cut into 4 μm sections. Afterward, the sections were stained with hematoxylin and eosin (H&E) according to the method described by Na, Li [78]. Morphological changes in the lung tissues were recorded by a Zeiss Axioscope microscope (Carl Zeiss QEC GmbH, Peine, Germany). Stained sections were evaluated according to the guidelines of the American Thoracic Society [79] and the lung injury scoring system was based on the neutrophils in the alveolar space, neutrophils in the interstitial space, hyaline membranes, proteinaceous debris filling the airspaces, and alveolar septal thickening.

### 4.11. Total RNA Isolation and qRT-PCR Analysis

An Aurum^TM^ Total RNA Mini Kit (7326820, Bio-Rad, Mississauga, ON, Canada) was used to isolate total RNA and purified according to the manufacturer’s instructions. The concentrations of RNA extracts were determined using a TECAN^TM^ Nano-Quant Plate (Tecan Trading AG, Mannedorf, Switzerland). The purity of the RNA samples was determined by ensuring the 260/280 nm absorbance ratio was 1.8 and above. cDNA was synthesized from the extracted RNA by using the iScript^TM^ cDNA Synthesis Kit (1708891, Bio-Rad, Mississauga, ON, Canada). Extracted RNA (1 µg) was mixed with nuclease-free water to give a volume of 15 µL, together with the 5× iScript Reaction Mix (4 µL) and iScript Reverse Transcriptase (1 µL) provided with the cDNA synthesis kit. This mixture (final volume 20 µL) was subjected to a thermal cycle of 25 °C for 5 min (priming), 46 °C for 20 min (reverse transcription), and 95 °C for 1 min (reverse transcriptase inactivation) to synthesize cDNA in CFX Connect^TM^ Real-Time System (Bio-Rad, Hercules, CA, USA). mRNA expression levels of the targeted genes; TLR4, ERK1, ERK2, JNK, NF-κB, cFOS, cJUN, IL-1*β*, IL-4, IL-6, and TNF were measured by using primers purchased from Bio-Rad Laboratories Ltd., Mississauga, ON, Canada. PCR amplification of the RNA regions targeted by the primers (Table 3) was performed by mixing the prepared cDNA (1/4 diluted, 2.5 µL) with primer (0.5 µL), nuclease-free water (2 µL), and 5 µL of SsoAdvanced^TM^ Universal SYBR^®^ Green Supermix (172-5274, Bio-Rad, Mississauga, ON, Canada). PCR amplification was conducted in a CFX Connect^TM^ Real-Time System (Bio-Rad, Hercules, CA, USA) by using a thermal cycle of 95 °C for 1 min and 40 repeated cycles of 95 °C for 15 s and 66 °C for 30 s. Data were collected and analyzed by the Bio-Rad CFX Maestro 2.3 software (Bio-Rad, Hercules, CA, USA). *β*-Actin and GAPDH were used as reference genes to normalize the expression of targeted genes. Results were expressed as normalized 2^−ΔΔCt^ relative to the negative control.

### 4.12. Western Blotting Techniques for Protein Expression

The harvested lung tissue samples were lysed using ice-cold RIPA buffer containing freshly added protease inhibitors (1 mM phenylmethylsulfonylfluoride, 10 μg/mL aprotinin, 5 μg/mL leupeptin, 1 mM dithiothreitol, 5 µg/mL pepstatin, and 10 μM phenylarsine oxide) for a total of 15 min before use. The lysates were collected at 14,000× *g* in a cold centrifuge for 10 min, while the Bradford [80] method was used for protein concentration determination. Protein samples were denatured by the loading buffer pack (New England BioLabs^TM^ Inc., Ipswich, MA, USA) and equalized using the sample buffer. Equal amounts of protein were loaded into 4–20% precast gel cassettes (Mini-PROTEANS^®^ TGX Stain-free gels, Bio-Rad Laboratories Inc., Hercules, CA, USA) and separated using SDS-PAGE electrophoresis for 1 h at 125 V and 400 mA, respectively. After proteins migrated based on molecular weight, they were transferred into polyvinylidene fluoride (PVDF) membranes and blots were incubated in 5% BSA prepared in TBST buffer (Tris-buffered saline, 0.1% Tween 20) for 1 h to block non-specific binding. Blots were then probed with primary antibodies overnight at 4 °C. Blots were further probed with HRP-conjugated donkey anti-rabbit or anti-mouse IgG antibody for an hour after being washed thoroughly with TBST. Uniform protein loading was confirmed by probing the blots with HRP-conjugated rabbit anti-actin antibody. Signals were captured by enhanced chemiluminescence (ECL) based on the Clarity^TM^ and Clarity Max^TM^ Western ECL substrates kit (Bio-Rad Laboratories Inc., Hercules, CA, USA) to confirm the proteins of interest. ImageJ software (version 1.54g, Bethesda, MD, USA) was used for Western blot quantitative analyses.

### 4.13. Statistical Data Analysis

Data were reported as mean values calculated from replicates of at least three independent studies. Statistical tests were carried out using XLSTAT software perpetual version 2019.2.2 (Addinsoft Inc., New York, NY, USA). The Student *t*-test was employed for comparing two conditions, and one-way analysis of variance (ANOVA) was used with Tukey’s post hoc test for more than two comparisons. Values with *p* ≤ 0.05 and 0.001 were taken as statistically significant.

## 5. Conclusions

The results of this investigation suggest that sea cucumber and wild blueberry extracts contain bioactive molecules that exhibit anti-inflammatory properties against ALI. These properties could link to the presence of saponins (frondoside A) in sea cucumber extract and polyphenols (primarily anthocyanins) in the wild blueberry extracts. The mechanism of action for the reduction in inflammation is postulated to be through the inhibition of inflammatory cytokine release. In this study, the combination of sea cucumber and wild blueberry extracts revealed a significant and synergistic reduction in varying degrees of ALI lesions such as distorted parenchyma, increased alveoli thickness, lymphocyte and neutrophil infiltrations, fibrin deposition, pulmonary emphysema, pneumonia, intra-alveolar hemorrhage, and edema. The results encourage the development and assessment of natural health products (NHP) using sea cucumber and wild blueberry to manage ALI.

## Figures and Tables

**Table 1:** The body weight parameters of mice with oral supplements of SC and WB.

Parameters	Groups	Days of Observation		Percentage (%)
		Day 0 (g)	Day 7 (g)	
**Body Weight (g)**	1—Control	25.04 ± 0.66	25.18 ± 0.69	0.52 ± 0.22
	2—LPS	26.03 ± 0.49	26.34 ± 0.46	0.85 ± 0.41
	3—LPS + BP	25.83 ± 0.35	25.59 ± 0.25	0.75 ± 0.29
	4—LPS + WB	24.28 ± 0.45	24.72 ± 0.46	1.84 ± 0.42 *
	5—LPS + BP + WB	24.56 ± 0.64	25.43 ± 0.59	3.85 ± 0.79 **

Data are presented as Mean ± SEM (*n* = 10). LPS = Lipopolysaccharide; SC = Body parts of Sea Cucumber; WB = Wild blueberry. Significance was taken at * *p* ≤ 0.05 and ** *p* ≤ 0.01.

**Table 2:** Rationale for dosage of the extracts used for dietary supplementation of C57BL/6 mice.

Source of Extract	Bioactive Biomarker Compound in the Extract	The Concentration of the Biomarker Compound in the Extract	Dosage of Extract (7 Days)	Reference Used for the Selected Animal Dose
Sea cucumber (Body parts/internal organs)	Frondoside A (Saponin)	0.36 mg frondoside A/g DW extract	27.7 mg extract/mouse/day	Adrian and Collin [34]
Wild blueberry	Anthocyanins (Polyphenol)	18.95 mg P3GE/g DW extract	50.7 mg extract/mouse/day	Sandoval-Ramírez, Catalán [73]

P3GE, petunidin-3-*O*-glucoside equivalents; DW, dry weight. The dietary supplementation of the sea cucumber and wild blueberry extracts was calculated based on the quantification of frondoside A using HPLC/ESI/MS and P3GE using pH differential methods while the dosage for the animal study was calculated based on previous studies [34,73].

**Table 3:** Primer sequences used for real-time PCR analysis.

Gene	Forward	Reverse	Accession No.
*GAPDH*	GGGAAGCCCATCACCATCTT	GCCTTCTCCATGGTGGTGAA	NM_008084.3
*ACTIN*	CTCTGGCTCCTAGCACCATGAAGA	GTAAAACGCAGCTCAGTAACAGTCCG	NM_007393.5
*NF-κB*	AAGAACAGAGACCGCTGGTG	CAGGTTCTGCATCCCCTCTG	XM_006509023.5
*IL-6*	CTGCAAGAGACTTCCATCCAG	AGTGGTATAGACAGGTCTGTTGG	NM_031168
*TLR4*	ACTCAGCAAAGTCCCTGATGACA	AGGTGGTGTAAGCCATGCCA	NM_021297.3
*p38 (MAPK14)*	GCCGCTTAGTCACATACCACT	GTCCCCGTCAGACGCATTAT	NM_001357724.1
*JNK1 (MAPK8)*	CTTCAGAAGCAGAAGCCCCA	TGTGCTAAAGGAGACGGCTG	NM_016700.4
*ERK1 (MAPK3)*	ACACTGGCTTTCTGACGGAG	TGATGCGCTTGTTTGGGTTG	NM_011952.2
*ERK2 (MAPK1)*	TTGCTTTCTCTCCCGCACAA	AGCCCTTGTCCTGACCAATTT	NM_011949.3
*Jun (AP-1)*	AAGAAGCTCACAAGTCCGGG	GAGGGCATCGTCGTAGAAGG	NM_010591.2
*FOS (AP-1)*	TGTTCCTGGCAATAGCGTGT	TCAGACCACCTCGACAATGC	NM_010234.3
*IL-1β*	GAAATGCCACCTTTTGACAGTG	TGGATGCTCTCATCAGGACAG	NM_008361
*TNF-α*	CAGGCGGTGCCTATGTCTC	CGATCACCCCGAAGTTCAGTAG	NM_013693
*IL-4*	CGACTGCACAGCAGTTCCA	CTCTGGTTGGCTTCCTTCACA	NM_172348

## Data Availability

Data are contained within the article.
